# AURKA and FAM83A are prognostic biomarkers and correlated with Tumor-infiltrating Lymphocytes in smoking related Lung Adenocarcinoma

**DOI:** 10.7150/jca.51321

**Published:** 2021-01-18

**Authors:** Mengyu Zhang, Chen Huo, Yingxiao Jiang, Jianyu Liu, Yican Yang, Yunhong Yin, Yiqing Qu

**Affiliations:** 1 Department of Pulmonary and Critical Care Medicine, Qilu Hospital, Cheeloo College of Medicine, Shandong University, Jinan 250012, China.; 2 Department of Pulmonary and Critical Care Medicine, Qilu Hospital of Shandong University, Jinan 250012, China.

**Keywords:** lung adenocarcinoma, smoking, prognosis, tumor-infiltrating lymphocytes

## Abstract

Lung adenocarcinoma (LUAD) has become the main histologic type, which account for nearly 40% of lung cancer. The present study aimed to investigate the gene expression signature in smoking related LUAD. A total of 45 smoking related DEGs in LUAD were identified and functional enrichment analysis was also performed. Then Cox's regression model and Kaplan-Meier analysis were used to screen potential prognostic genes. Finally, AURKA and FAM83A were left for further immune-related mechanism exploration. Kaplan-Meier analysis indicated survival rates are related to different immune cell (B cell and Dendritic cell) infiltration levels. Mechanistically, we further explore the correlation between AURKA and FAM83A gene expression levels and tumor-infiltrating lymphocytes (TILs) level as well as their response to immunomodulators. The results suggested that AURKA and FAM83A are highly expressed in smoking related LUAD, and negatively correlated to B cell and Dendritic cell infiltration levels. At the same time, B cell and Dendritic cell infiltration levels also related to the prognosis of LUAD. We further revealed AURKA and FAM83A could be novel targets to improve the prognosis of LUAD through regulated the response to immunomodulators.

## Introduction

Lung cancer is still the most common leading cause of cancer death worldwide 1. This differs from other cancers, less than 50% of lung cancer patients only could survive one year after diagnosed and the 5-year survival rate is only 18% at present 2,3. Lung adenocarcinoma (LUAD) has become the main histologic type, which account for nearly 40% of lung cancer 3,4. It is known that cigarette smoke is the most important risk factor, which leads to the majority of lung cancers. LUAD usually happened in the distal airway and frequently observed in smokers 5, while patients those abstain from smoking have a better prognosis than those continuing smokers after diagnosis 6.

Cigarette smoke consists of various chemicals which could cause badly damage to the respiratory epithelium as well as its genome exchange 7. It is already studied that smoking history could result in higher frequencies of genomic alteration in lung cancer 8,9. Besides, tumor-infiltrating lymphocytes (TILs) has been demonstrated to be prognostic biomarkers in NSCLC 10,11. However, the role of TILs as potential prognostic biomarkers remains controversial because elevated levels of TIL has been associated with both better and worse prognosis in NSCLC patients 12-15. Previous studies demonstrated that the prognostic value of TILs significantly different according to histological type and smoking habit in NSCLC patients 16, which suggests that TILs may play key roles in lung cancer.

However, the exact relationships between gene expression signature and TILs levels in LUAD have not been well elucidated according to the status of smoking. Therefore, it is vital to elucidate the gene expression changes and TILs levels in the diagnosis and prognosis of LUAD according to the smoking history. In this study, we identified smoking related gene expression signature and explored their correlation with TILs in LUAD.

## Materials and Methods

### Data collection and identification of DEGs

Two smoking related LUAD gene expression profiles (GSE31210 and GSE43458) were retrieved from the Gene Expression Omnibus (GEO) database (http://www.ncbi.nlm.nih.gov/geo/). In brief, GSE31210 included 111 smoking related LUAD tissues and 115 non-smokers. GSE43458 included a total of 80 LUAD tissues which were comprised of comprised of 40 smokers and 40 non-smokers. The selected criteria were the followings: gene expression profiles; studies compared genes expression between smoker and non-smoker LUAD tissues in human. The excluded criteria were the followings: those studies that compared genes between lung cancer and adjacent non-tumorous lung tissues or normal tissues or benign disease in human; expression profiles using cell lines or serum, saliva, peripheral blood; studies did not include status of smoking; patient had no survival time or survival status; patient had clinical information but no gene expression data. GEO2R (https://www.ncbi.nlm.nih.gov/geo/geo2r/) are used to screen DEGs by comparing LUAD tissues between smoker and non-smoker groups. The criterion of DEGs were defined as P < 0.01 and the absolute fold change > 1.5. Besides, several R packages were also used to visualized the data. The ggplot2 package (https://cran.r-project.org/web/packages/ggplot2) in R software was used to perform the volcano plots in 2 smoking related LUAD GEO datasets; Venn Diagram package (https://cran.r-project.org/web/packages/VennDiagram/) was applied to identify the overlapped DEGs in 2 smoking related LUAD GEO datasets. Pheatmap package (https://cran.r-project.org/web/packages/pheatmap) was used to performed heat maps for overlapped 45 DEGs.

### Functional enrichment analysis

Functional enrichment comprised of KEGG pathway and GO enrichment analysis. In concise, KEGG pathway enrichment analysis could provide several signaling pathways associated with given genes, GO enrichment analysis could predict the biological functions of the target genes. In our study, GO and KEGG pathway enrichment analysis were performed by R software according to the Database for Annotation, Visualization, and Integrated Discovery (DAVID) (https://david.ncifcrf.gov/) results. In this category, P<0.05 was the cutoff value to identify significant GO terms and KEGG pathways.

### TCGA datasets and identification of prognosis-related genes

The gene expression profiles and clinical data of LUAD patients were downloaded from the TCGA data portal (https://tcgadata.nci.nih.gov/tcga/). Finally, a total of 500 smoking related LUAD tissues, including 159 non-smokers and 341 smokers were used to further prognostic-related analysis. Detailed criteria for the inclusion and exclusion of patients have been presented in the “Data collection and identification of DEGs” part.

Cox's regression model, also named as proportional hazards model, uses survival outcomes and survival time as dependent variables. This model not only could analyze the impact of many factors on survival at the same time, also can analyze data with censored survival time and does not require estimation of the survival distribution type of the data.

In this study, Cox's regression model was used to assess the prognostic value of 45 DEGs. Firstly, univariable Cox regression analysis was performed using the survival package in R software and 12 DEGs were identified for next analysis (P<0.05). Then, 12 DEGs were further analyzed by stepwise multivariate Cox regression analysis. During the analysis procedure, Akaike information criterions (AICs) was used to select the lowest AIC value as a predicative model as well as the best performance efficacy predictive model. Finally, a total of four genes (AURKA, FAM83A, HSD17B2 and POU2AF1) were identified as prognostic genes.

### Validation of prognostic genes

Prognostic genes were validated using public database Gene Expression Profiling Interactive Analysis (GEPIA) (http://gepia.cancer-pku.cn) and Kaplan Meier-plotter (https://kmplot.com/analysis/). GEPIA database contain expression data of various tumor samples as well as normal samples, including LUAD. Kaplan Meier-plotter is an online tool to perform survival curves, in which contain clinical data such as survival time and smoking history of various tumors. These two databases were used to verify the prognostic value of the DEGs.

### GSEA analysis

GSEA analysis was used to further understand AURKA-related as well as FAM83A-related pathways. AURKA and FAM83A expression levels were used to grouping the LUAD patients in an independent TCGA cohort, samples with the expression levels were higher than the median value of both AURKA and FAM83A expression were assigned to the high expression group, rest of the samples are defined as low expression group automatically. The collection of annotated gene sets of c2.cp.kegg.v6.2.symbols.gmt was used to the reference gene sets and GSEA software was used to preform GSEA analysis. The cut-off criterion is FDR< 0.01.

### Explore the immune-related mechanism of AURKA and FAM83A

Immune-related mechanism of AURKA and FAM83A were performed by TISDIB (http://cis.hku.hk/TISIDB/) and Tumor IMmune Estimation Resource TIMER (https://cistrome.shinyapps.io/timer/). In brief, TISIDB is an integrated repository portal for tumor-immune system interactions. It is also a powerful website containing a large amount of tumor immunity-related data, which is conducive to comprehensive research on the interaction between tumor and immunity. TIMER web server is a comprehensive resource for systematical analysis of immune infiltrates across diverse cancer types. The abundances of six immune infiltrates (B cells, CD4+ T cells, CD8+ T cells, Neutrophils, Macrophages, and Dendritic cells) are estimated by TIMER algorithm. TIMER web server allows users to input function-specific parameters, with resulting figures dynamically displayed to conveniently access the tumor immunological, clinical, and genomic features.

In this study, we performed TISIDB-Reactome pathway analysis of AURKA and FAM83A. Besides, we also validate AURKA and FAM83A expression levels and their relation to OS in both TISDIB and TIMER database. We further explored cumulative survival rates between low and high immune cell (B Cell and Dendritic Cell) infiltration levels in TIMER. Then correlation between AURKA as well as FAM83A expression level and immune cell infiltration level were explored, so as the correlation between SCNA levels of two genes and immune cell infiltration level. Finally, gene expression levels and SCNA levels of two genes and their relationships to immunoinhibitors also examined. Purity-corrected partial Spearman method was used to analyze the data.

### Statistical analysis

Statistical analyses were performing using SPSS IBM for windows version 23.0 (IBM Corporation, Armonk, NY, USA) and Graph Pad Prism 7.0 (GraphPad Software, Inc., La Jolla, CA, USA). We divided patients into the high and low expression of AURKA and FAM83A groups with the median of gene expression levels. Single comparison of the expression rates between two groups were determined by Student's t-test. Kaplan Meier survival curves were performed by TCGA cohort and examined by Log-rank test. The correlation between gene expression levels and infiltrating immune cell levels are used purity-corrected partial Spearman method. P<0.05 was considered statistically significant.

## Results

### Identification of differentially expressed genes (DEGs) and functional enrichment in smoking related LUAD

**Figure 1:**
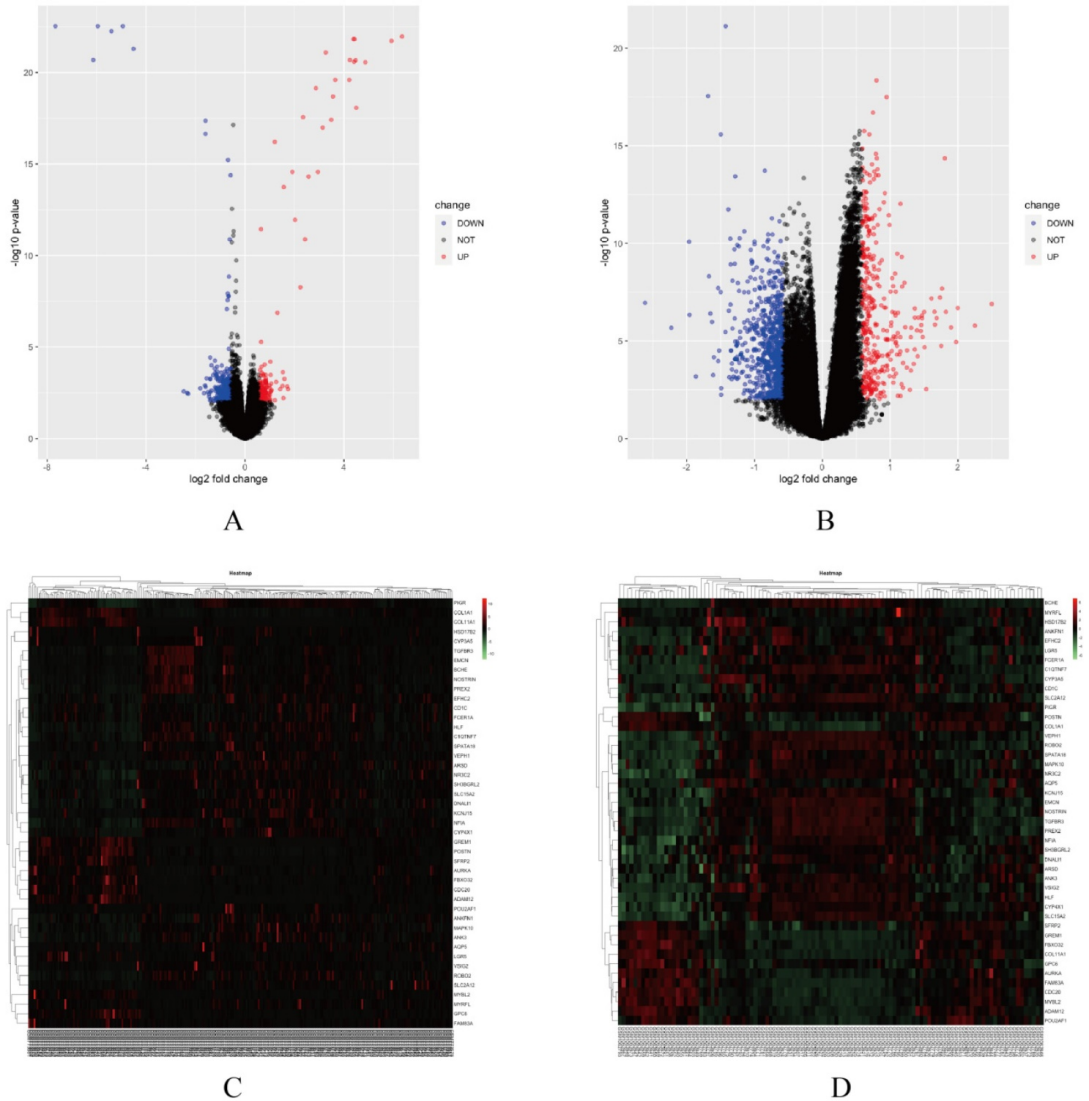
** Screening differentially expressed gene signature.** (A-B) Volcano plots of the gene expression signature analysis in GSE31210 and GSE43458. (C-D) Heatmaps of 45 DEGs expression levels in GSE31210 and GSE43458.

**Figure 2:**
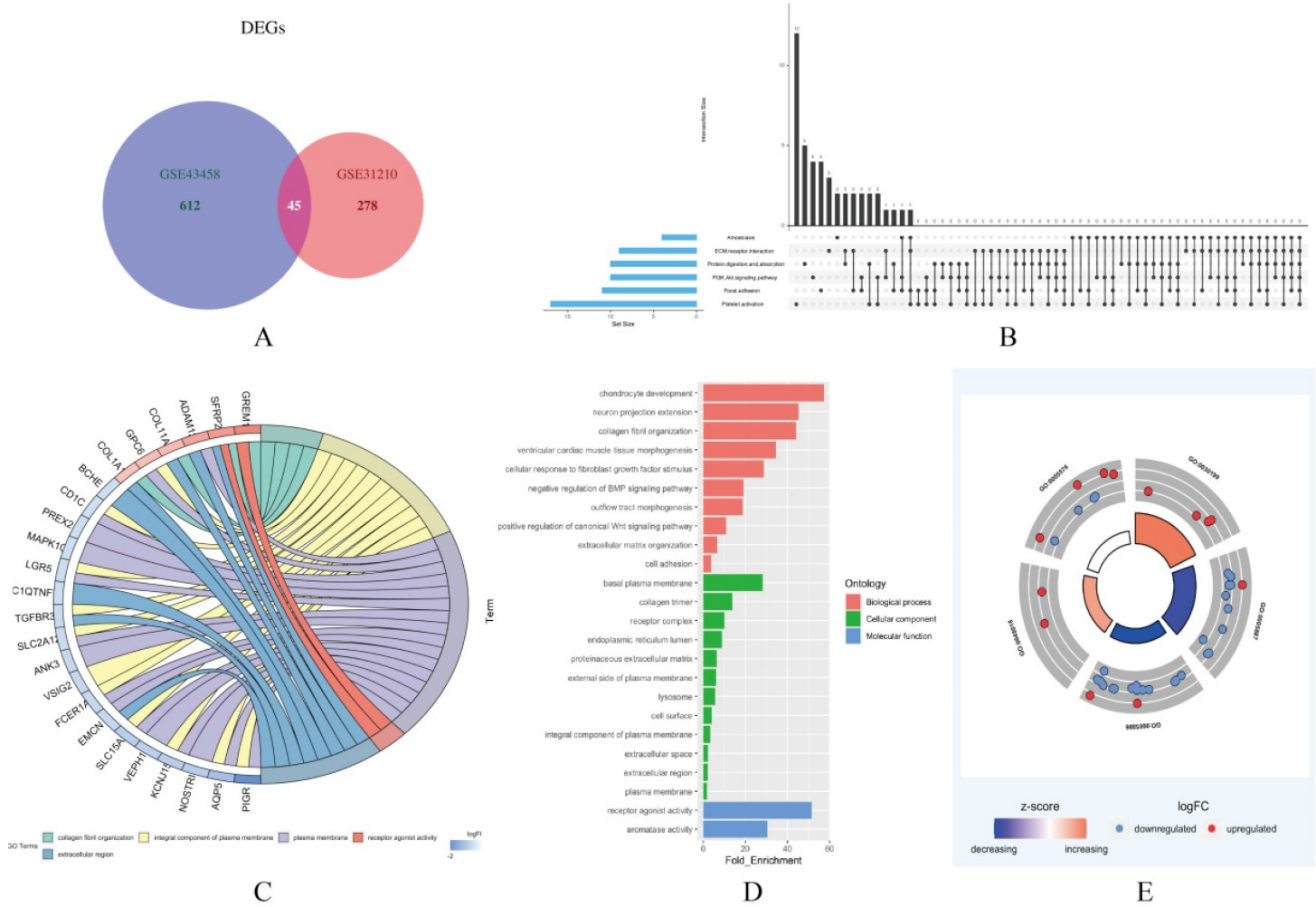
**Functional enrichment analysis of 45 DEGs in smoking related LUAD.** (A) A total of 45 genes were overlapped in two smoking related LUAD GEO datasets (GSE31210 and GSE43458). (B) UpSet view of enriched KEGG pathways in LUAD. X-axis: gene number of pathway types. Y-axis: the number of genes for one or several pathway types. (C) The circle plots showed the gene ontology terms and their correlation among top 24 genes. (D) The significantly enriched gene ontology terms. (E) The outer circle indicated the expression of DEGs. The inner circle indicated the significantly enriched GO terms.

In this study, two smoking related LUAD gene expression profiles were extracted from GEO database to explore gene expression signature, including GSE31210 and GSE43458. GSE31210 including 111 smokers and 115 non-smokers, GSE43458 including 40 smokers and 40 non-smokers. Genes with P<0.01 and absolute fold change>1.5 were defined as DEGs. After screening process, there are 323 genes differentially expressed in GSE31210 (Figure 1A) and 657 DEGs in GSE43458 (Figure 1B). After overlapping DEGs between GSE31210 and GSE43458, 45 DEGs were identified in smoking related LUAD patients (Figure 2A). For further visualization, we also performed heatmaps of 45 DEGs in both GSE31210 and GSE43458 (Figure 1C and 1D). Finally, 45 DEGs were screened in smoking related LUAD patients. To explore the biological functions of the 45 DEGs, gene ontology (GO) and Kyoto Encyclopedia of Genes and Genomes (KEGG) pathway analysis were performed. The GO analysis results showed these 45 DEGs were mainly involved in chondrocyte development, basal plasma membrane and receptor agonist activity in biological process, cellular component and molecular function, respectively (Figure 2C-2E). Besides, KEGG pathway analysis demonstrated that most of these target genes were enriched in PI3K/Akt signaling pathway (Figure 2B). All the results suggest that changes in immune and metabolic-related are pathways are also necessary for tumorigenesis and development.

### Prognostic gene expression signature was identified in smoking related LUAD

**Figure 3:**
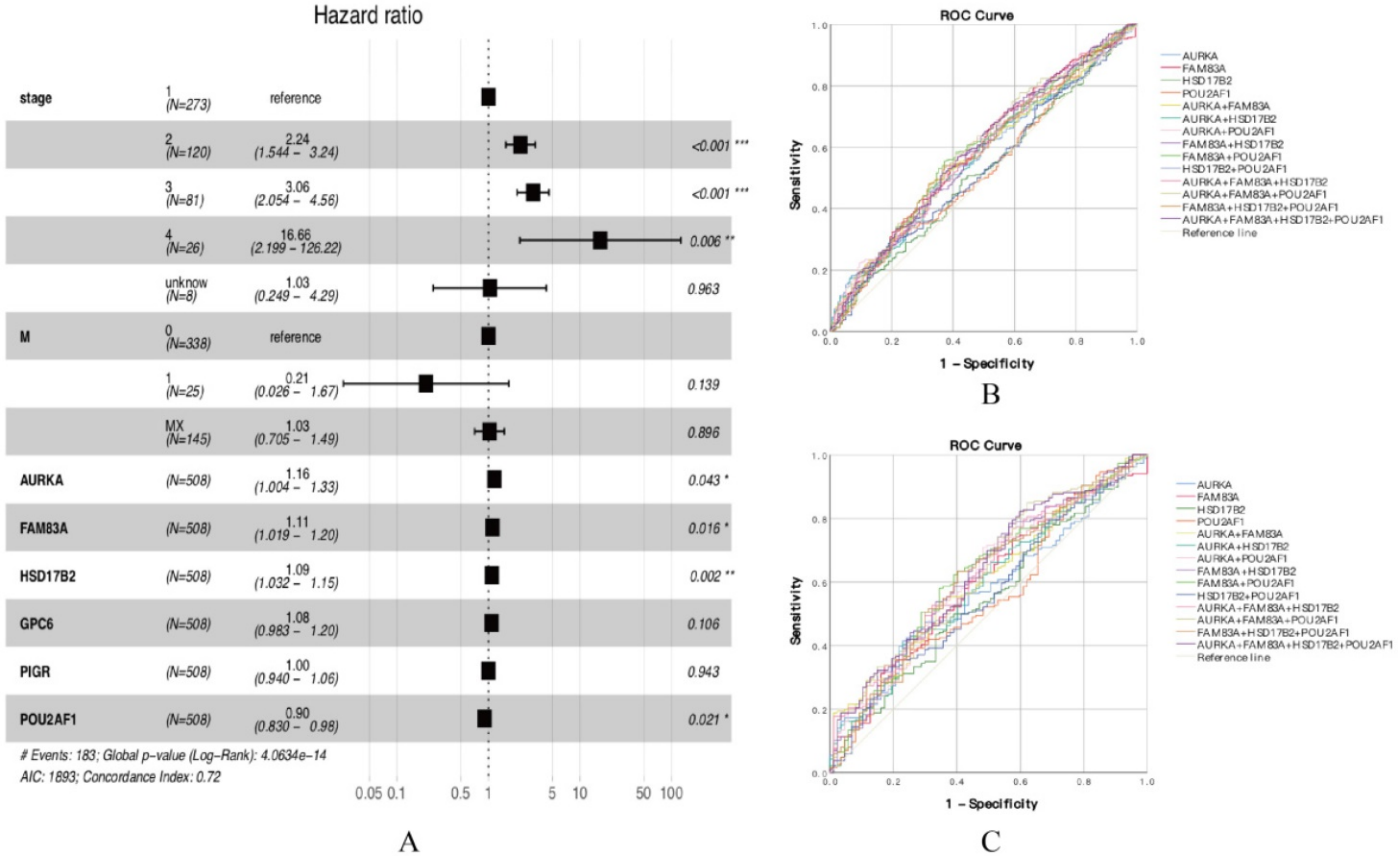
(A) Forest plot of multivariate cox regression analysis. (B) ROC analysis of four DEGs and the combination diagnostic value of LUAD patients. (C) ROC analysis of four DEGs and the combination diagnostic value of early stage LUAD patients (Stage I).

According to the functional enrichment analysis results, 45 DEGs were considered to have a significant connection with tumorigenesis and development, so we further explored the prognostic value of 45 DEGs in smoking related LUAD. Univariate Cox proportional hazards regression analysis was performed to identify the significant prognostic genes, then all the significant prognostic genes were used to perform multivariate Cox proportional hazards regression analysis. The univariate Cox proportional hazards regression analysis showed that there are 12 genes have prognostic values (Table 1). Multivariate Cox proportional hazards regression analysis of the 12 genes revealed that AURKA, FAM83A, HSD17B2 and POU2AF1 have prognostic values (Figure 3A, Table 2). The results showed that the hazard ratio (HR) of AURKA was 1.156 with 95% confidence interval (CI) is 1.004-1.331 (P=0.043), the HR of FAM83A was 1.107 with 95% CI is 1.019-1.202 (P=0.016), the HR of HSD17B2 was 1.091 with 95% CI is 1.032-1.154 (P=0.002) and the HR of POU2AF1 was 0.904 with 95% CI is 0.830-0.985 (P=0.002), which means that AURKA, FAM83A and HSD17B2 are risk factors, whereas POU2AF1 is a protective factor. Besides, we also performed receiver operating characteristic (ROC) curve analysis to estimate the diagnostic value of 4 genes using an independent LUAD cohort from TCGA database, in which including 341 smokers and 159 non-smokers. Unfortunately, the results showed that the area under curve (AUC) of all the groups seems have no significant difference in all LUAD patients (Figure 3B, Table S1). Besides, the same results also showed at early stage LUAD patients (Stage I) (Figure 3C, Table S2), in which including 186 smokers and 87 non-smokers.

### Association of AURKA, FAM83A, HSD17B2 and POU2AF1 expression levels with overall survival (OS) of smoking related LUAD

**Figure 4:**
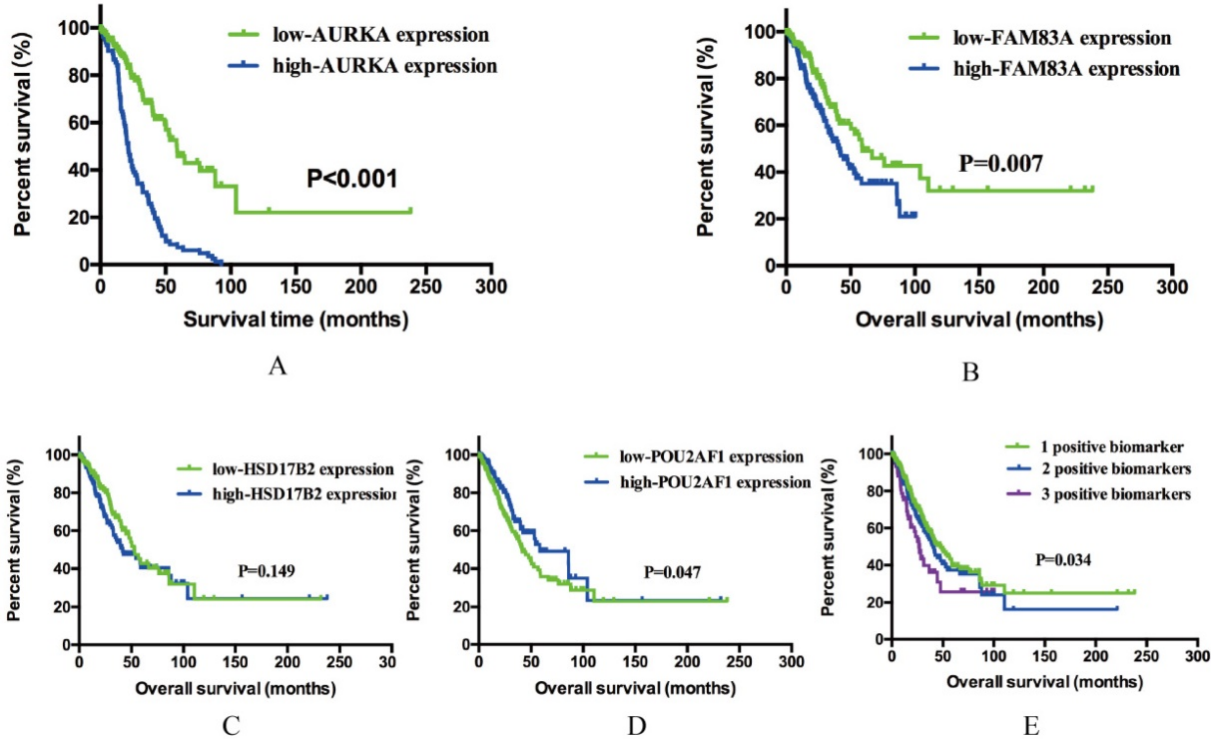
** Kaplan-Meier survival analysis by different gene expression levels of AURKA, FAM83A, HSD17B2 and POU2AF1 in 341 smoking related LUAD patients from an independent TCGA cohort.** (A) OS between low and high AURKA expression. (B) OS between low and high FAM83A expression. (C) OS between low and high HSD17B2 expression. (D) OS between low and high POU2AF1 expression. (E) OS among three different groups.

Previous results demonstrated that AURKA, FAM83A, HSD17B2 and POU2AF1 may have prognostic values in smoking related LUAD. To further explore whether these four genes will affect the clinical outcomes, we performed Kaplan-Meier analysis using TCGA cohort. As shown in Figure 4A-D, AURKA expression was significantly associated with OS (P<0.001) among the smoking related LUAD. The median OS in AURKA low expression group was 58.80 months whereas in high expression group was 21.41 months. Similarly, higher expression of FAM83A is associated with a shorter OS (P=0.007), the median OS was 59.07 months in low expression group and 41.56 months in high expression group. As for POU2AF1, its low expression was remarkably related to longer OS (P=0.001). The median OS in low expression group was 58.41 months, while in high expression group was 41.56 months. While speaking about HSD17B2, its expression level seems have no significance with OS statistically (P=0.149).

Based on previous OS analysis results, there are three significant prognostic genes, further to assess the integrated effects of these three genes on the prognosis, we divided all these smoking related LUAD patients into three groups according to the number of positive biomarkers. Positive biomarkers defined as high expression of AURKA and FAM83A as well as the low expression of POU2AF1. Group 1 have any one positive biomarker of three genes, group 2 have any two positive biomarkers of three genes and group 3 have all these three biomarkers. The results revealed that positive biomarker numbers indeed associated with OS (P=0.034), which means that united of 3 genes maybe forecast the OS of smoking related LUAD (Figure 4E). In conclusion, high expression of AURKA and FAM83A as well as the low expression of POU2AF1 can be used as potential prognostic biomarkers in smoking related LUAD patients.

### Validation of AURKA, FAM83A and POU2AF1 expression levels and their relation with OS in LUAD

**Figure 5:**
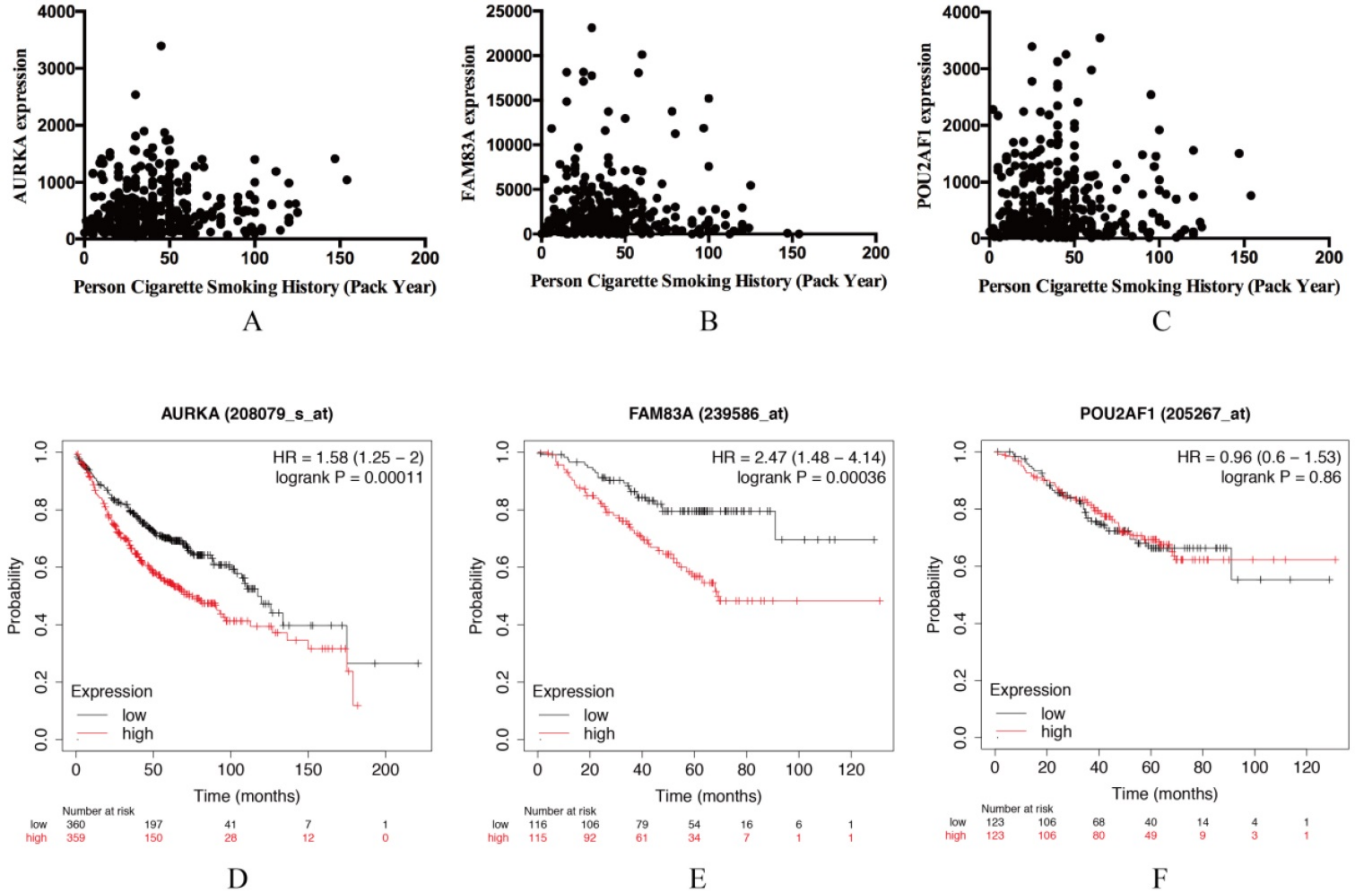
(A-C) The relationship between AURKA, FAM83A, POU2AF1 expression levels and person cigarette smoking history (pack year). (D-F) Kaplan-Meier survival analysis of AURKA, FAM83A, POU2AF1 in smoking-related LUAD according to Kaplan Meier-plotter.

**Figure 6:**
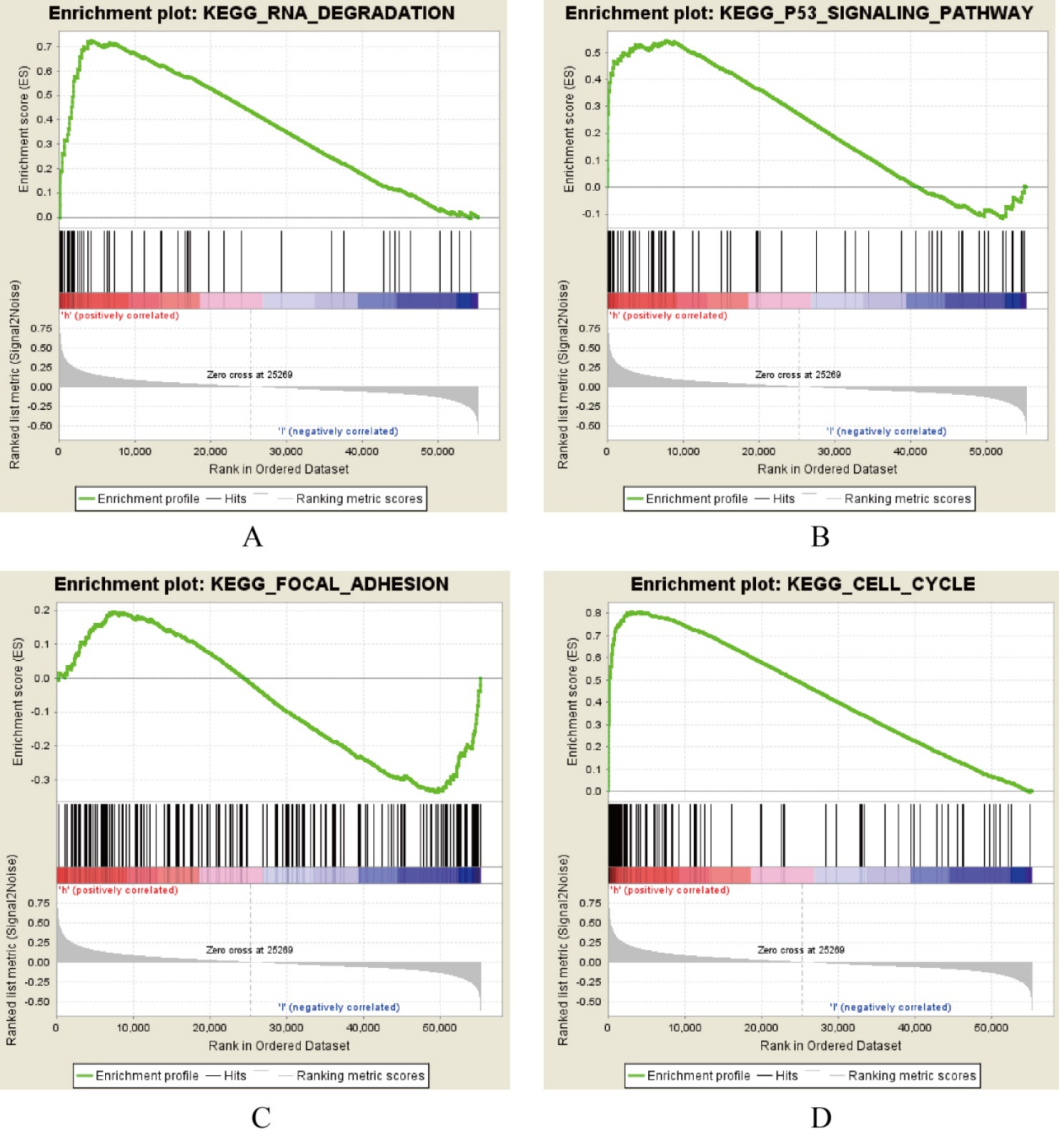
AURKA and FAM83A correlated enrichment gene analysis with GSEA.

AURKA, FAM83A and POU2AF1 were identified as prognostic genes in TCGA database, we also validated their expression levels in online tool GEPIA. Consistent with previous results, AURKA, FAM83A and POU2AF1 were highly expressed in LUAD patients compared with normal controls (Figure S1A-C). Besides, we also explored their expression levels among different stages of LUAD patients and the results suggested that their expression level were also related to stage (Figure S1D-F). Furthermore, we also performed Kaplan-Meier analysis using Kaplan Meier-plotter online tool to validate the prognostic value of AURKA, FAM83A and POU2AF1. However, different with our previous results, POU2AF1 expression level seems have no effects on OS in smoking related LUAD (Figure 5D-6F). Considering smoking history of LUAD patients, we explored the relationship between AURKA, FAM83A, POU2AF1 expression levels and person cigarette smoking history (pack year). Different with our expectation, it seems that there is no relation between AURKA, FAM83A, POU2AF1 expression levels and person cigarette smoking history (pack year) statistically (P=0.150, P=0.592A and P=0.724, respectively) (Figure 5A-6C). Finally, we analyzed the pathway of high-expression of AURKA and FAM83A by GSEA analysis. The results showed that high AURKA and FAM83A expression samples were mainly enriched in RNA degradation, P53 signaling pathway, focal adhesion and cell cycle (Figure 6).

### Associations of AURKA and FAM83A expression levels with clinicopathological variables

Clinicopathological characteristics of the smoking related lung adenocarcinoma patients are listed in Table 3. As Table 3 showed, AURKA expression was remarkably positively associated with gender (P<0.001), smoking history (P=0.016), pathological T stage (P=0.029) and pathological N stage (P=0.032). No significant difference of AURKA mRNA levels was found in patients with age (P=0.277) and pathological M stage (P=0.437); the elevated FAM83A expression was closely related with gender (P=0.019), smoking history (P=0.001), pathological T stage (P=0.010) and pathological N stage (P=0.004).

### AURKA and FAM83A expression levels and associations between TILs and OS

**Figure 7:**
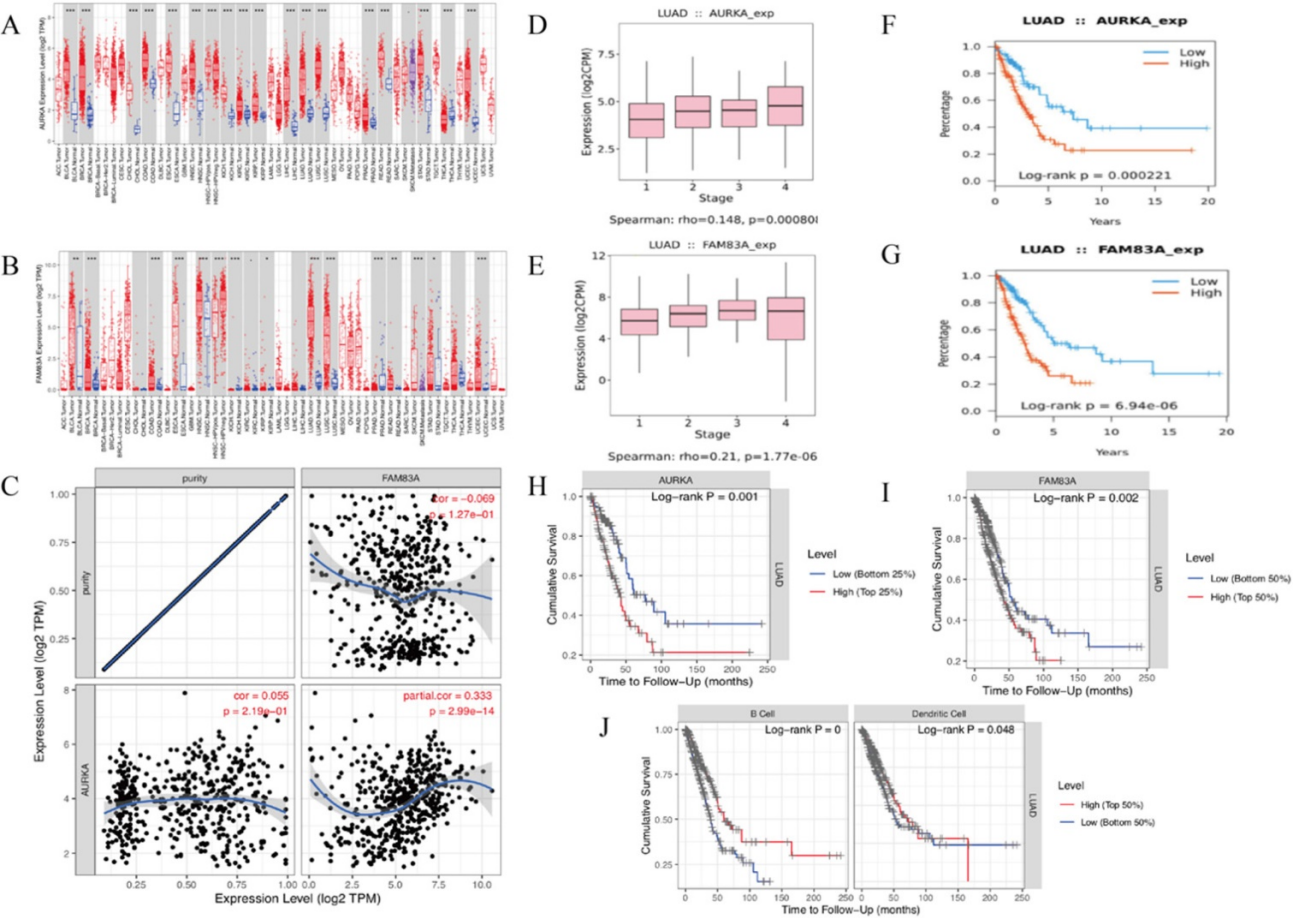
(A-B) AURKA and FAM83A expression levels in various cancer and normal tissues. (C) Correlation between AURKA and FAM83A expression levels as tumor purity increasing. (D-E) AURKA and FAM83A expression levels in different stages of LUAD. (F-G) Kaplan-Meier survival analysis by different AURKA and FAM83A expression levels of LUAD. (H-I) Kaplan-Meier survival analysis by different AURKA and FAM83A expression levels of LUAD. (J) Cumulative survival rates between low and high immune cell (B Cell and Dendritic Cell) infiltration.

The KEGG pathway and Gene set enrichment analysis (GSEA) enrichment analysis results showed that AURKA and FAM83A are hub genes of cancer-related pathways. TISIDB-Reactome pathway analysis shows the similar results that AURKA and FAM83A are involved in the cell cycle in the immune system (Table 4). Firstly, we validate AURKA and FAM83A expression levels (Figure 7A-B) as well as their expression levels in different stages (Figure 7D-E) and their relation to OS in both TISIDB and TIMER databases (Figure 7F-I). Furthermore, correlation between AURKA and FAM83A expression levels were also explored. The results showed that as tumor purity increasing, AURKA and FAM83A expression levels are positive related (r=0.333, P=2.99e-14) (Figure 7C). Finally, we constructed Kaplan-Meier analysis between high and low TILs expression levels. Kaplan-Meier analysis results showed that cumulative survival rates between low and high immune cell (B cell and Dendritic cell) infiltration levels are different (P<0.001 and P=0.048) (Figure 7J).

**Figure 8:**
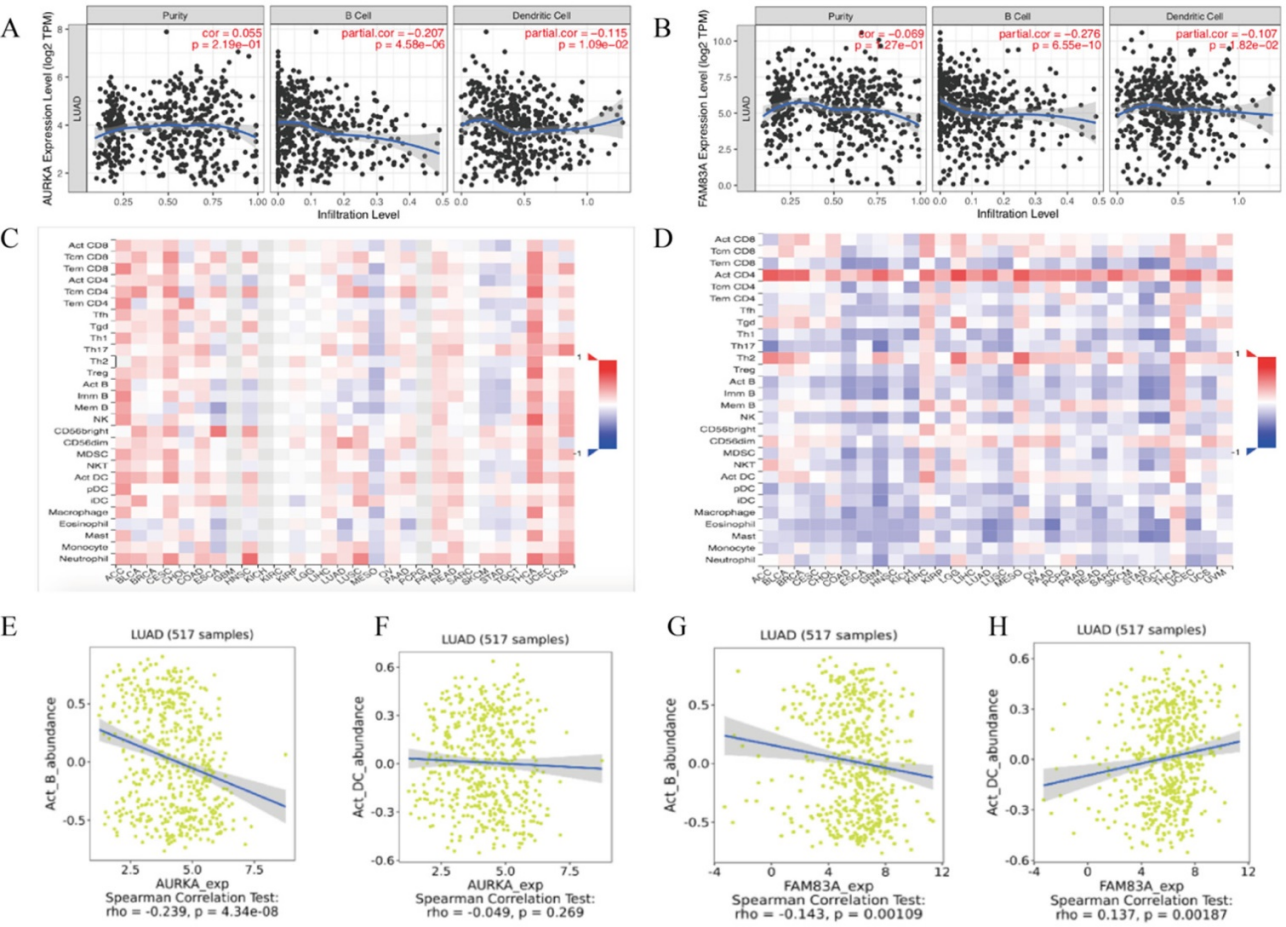
(A) Correlation between AURKA expression level and immune cell (B Cell and Dendritic Cell) infiltration level. (B) Correlation between FAM83A expression level and immune cell (B Cell and Dendritic Cell) infiltration level. (C-D) Heatmap analysis of AURAK and FAM83A expression levels and tumor infiltrating lymphocytes. (E) Correlation between AURKA expression level and B Cell infiltration level. (F) Correlation between AURKA expression level and Dendritic Cell infiltration level. (G) Correlation between FAM83A expression level and B Cell infiltration level. (H) Correlation between FAM83A expression level and Dendritic Cell infiltration level.

For further exploration, we conducted an integrated analysis to predict the potential biological roles of AURKA and FAM83A in tumor-immune of LUAD. The results showed that AURKA and FAM83A expression levels are negatively correlated to B cell (r=-0.207, P=4.58e-06; r=-0.276, P=6.55e-10) as well as Dendritic cell (r=-0.115, P=1.09e-02; r=-0.107, P=1.82e-02) infiltration levels in TIMER database (Figure 8A-B). Heatmap analysis between AURAK and FAM83A expression levels and tumor infiltrating lymphocytes in TISDIB database were also performed (Figure 8C-D). According to the heatmap analysis results, we found that B cell and Dendritic cell infiltration levels were negatively related to AURKA and FAM83A expression levels, so we also validated the correlation between AURKA and FAM83A expression levels and immune cell infiltration levels in TISDIB database. The results showed that AURKA and FAM83A expression levels are both negatively related to B cell infiltration level (r=-0.239, P=4.34e-08; r=-0.143, P=0.001), which consistent with previous results. As to dendritic cell infiltration level, it only positively related to FAM83A expression level (r=0.137, P=0.002), not AURKA expression level (r=-0.049, P=0.269), which differ from previous results.

### Associations between somatic copy number variation (SCNA) levels of AURKA and FAM83A and TILs levels

The ''SCNA'' module provides the comparison of the abundance of TILs among tumors with different somatic copy number aberrations for a given gene, we further explored the association between SCNA levels of AURKA and FAM83A with immune cell infiltration, respectively. The results indicated that copy number appears to correlate with AURKA and FAM83A expression in LUAD (Figure S2A-B). Heatmap analysis between SCNA levels of AURAK, FAM83A expression levels and TILs in TISDIB database were also performed (Figure S2C-D). Furthermore, SCNA levels of AURKA and FAM83A were also negatively correlated with B cell and Dendritic cell infiltration levels (both r<0; both P<0.05) (Figure S2E-H).

Previous studies have found that decreased AURKA and FAM83A expression could exhibited significant response of immunomodulators. So, to identify the expression patterns of AURKA and FAM83A response to immunomodulators, the heatmap analysis was performed (Figure S3A-H). Besides, we also explored the AURKA and FAM83A expression distribution across LUAD subtype (Figure S3I-J). Furthermore, TP53 mutation as the most type of LUAD mutation was related to CD8+ T cell, Neutrophil and Dendritic Cell infiltration levels (Figure S3K).

## Discussion

In this study, we firstly screened 45 DEGs in smoking related LUAD and performed functional enrichment analysis. Then according to an independent cohort from TCGA database, we performed a Cox's regression model and identified four prognosis-related genes. While after we validate their expression levels and their relation to OS in GEPIA and Kaplan Meier-plotter databases, there are only AURKA and FAM83A left for further immune-related mechanism exploration. Kaplan-Meier analysis indicated survival rates are related to different immune cell (B cell and dendritic cell) infiltration levels, so we further explore the correlation between these two gene expression levels and immune cell infiltration levels as well as their response to immunomodulators. The results suggested that, AURKA and FAM83A are highly expressed in smoking related LUAD, and negatively correlated to B cell and Dendritic cell infiltration levels, at the same time, B cell and Dendritic cell infiltration levels also related to the prognosis of LUAD. Besides, AURKA and FAM83A maybe could improve the prognosis of LUAD through regulated the response to immunomodulators.

Aurora kinase A (AURKA) is a putative low-penetrance tumor susceptibility gene in cell cycle regulation and centrosomal function 17, which is essential for centrosome function and maturation, spindle assembly, chromosome alignment, and mitotic entry 18. AURKA had been well studied in many cancers, such as gastrointestinal cancer 19, colorectal cancer 20, breast cancer 21, bladder cancer 22 as well as lung cancer 23,24. Recent studies indicated AURKA is associated with resistance to EGFR inhibitors in EGFR-mutant LUAD 25, and increased AURKA expression is associated with poor prognosis of NSCLC patients 26. Besides, AURKA inhibitor had been proved to anticancer therapy, including VX-680 27, Hesperidin 28, AZD1152 29 and MLN8237 30, while they still need to be approved for clinical use by the food and drug administration (FDA). Family with sequence similarity 83 (FAM83A), located on chromosome 8q24, which is the smallest member of the FAM83 family 31. FAM83A have been reported related to breast cancer 32, pancreatic cancer 33, hepatocellular carcinoma 34, ovarian cancer 35, especially in lung cancer 36. Recently, FAM83A was identified as survival‑related gene of LUAD 37, 38. A newly released study demonstrated that FAM83A could affects PD-L1 expression levels in LUAD cell and could be a biomarker for immune escape and PD-1/PD-L1- targeted therapy 39. Another study claimed that the effect of FAM83A proteins mutation is prevent them from binding to CK1, which will regulate cell division and apoptosis and lead to cancer development 40. Although there are many researches discussed about the function of AURKA and FAM83A, there is barely any research explore the relation between their expression and immune cell infiltration levels.

It is important that TILs are related to prognosis of several cancers, including colorectal cancer 41, ovarian cancer 42, breast cancer 43 as well as lung cancer 44. Besides, the significance of TILs on the prognosis of many cancer types has been examined. TILs could be affected by many factors. There is one study elucidated the epidural anaesthesia could preserves immune function and affect TILs 45. According to another study, researchers found that tumor areas with CD4+ and CD8+ lymphocytes have a better prognosis 46. High expression level of CD45RO+ TILs are related to a better OS in LUAD 47. Furthermore, AURKAi is reported to promote the recruitment of TILs in melanoma cells 48. There is also another study suggested that the therapeutic relevance of AURKA could be an immunotherapy target 49. While the relationships between AURKA, FAM83A expression and TILs expression level remains unclear. In the present study, we identified that tumor-infiltrating B cells and dendritic cells are negatively correlated to the AURKA and FAM83A expression levels, which could affect the prognosis of smoking related LUAD.

Lymphocyte infiltration has been identified as potential prognostic biomarkers during the tumor progression. More and more evidence suggested that tumor-infiltrating B cells could affect clinical outcomes among various cancers 50. There is one study indicated that B cell-derived lymphotoxin could promote cancer progression in prostate cancer through activate I kappa B kinase (IKK)-α and activator of transcription 3 (STAT3) signaling 51. Besides, tumor-infiltrating B cells have inhibited tumor progress by activating CD8+ T cells 52. Dendritic cells (DCs) play critical roles and initiated primary T-cell responses 53. Several studies suggested that infiltrating DCs distributed in NSCLC tumor areas 54-56. Meanwhile, the association between infiltrating DCs levels and smoking related LUAD have not been identified 55. So, we also explored the correlation between these two gene expression levels and immune cell infiltration levels as well as the response to immunomodulators.

## Conclusions

In conclusion, our present study suggested that AURKA and FAM83A are elevated in smoking related LUAD tissues. Besides, their overexpressed levels have a worse prognosis in smoking related LUAD. Furthermore, we also investigated the relationship between AURKA, FAM83A expression levels and TILs. The results indicated that AURKA and FAM83A were negatively correlated to B cell and Dendritic cell infiltration levels. However, it is still important to further explore the molecular mechanisms of AURKA and FAM83A contributing to smoking related LUAD through TILs in the future.

## Supplementary Material

Supplementary figures and tables.

## Figures and Tables

**Table 1:** Univariate Cox regression of 12 DEGs

id	HR	HR.95L	HR.95H	pvalue
C1QTNF7	0.996589659	0.994372685	0.998811576	**0.002642831**
AURKA	1.000329891	1.000110275	1.000549555	**0.003237107**
FAM83A	1.000089959	1.000066475	1.000113444	**6.00E-14**
DNALI1	0.999526883	0.999225391	0.999828467	**0.002108691**
HSD17B2	1.000313219	1.000123282	1.000503192	**0.001227937**
GPC6	1.000235328	1.00005201	1.00041868	**0.011866257**
NR3C2	0.999125808	0.998476792	0.999775245	**0.008340564**
PIGR	0.999993616	0.999987576	0.999999656	**0.038316977**
ROBO2	0.998272667	0.99703047	0.999516412	**0.006501188**
POU2AF1	0.999693419	0.999424571	0.99996234	**0.025457096**
SPATA18	0.999334389	0.998792412	0.99987666	**0.016145524**
SLC15A2	0.999557146	0.999207719	0.999906695	**0.013027323**

**Table 2:** Multivariate Cox regression of 12 DEGs

Gene symbol	HR	HR.95L	HR.95H	pvalue
C1QTNF7	0.998692803	0.995057196	1.002341694	0.482074983
AURKA	1.156331238	1.004476772	1.33114271	**0.043160974**
FAM83A	1.106931346	1.018960025	1.20249762	**0.016193837**
DNALI1	0.999843658	0.99950009	1.000187345	0.372573322
HSD17B2	1.091198182	1.032164985	1.153607699	**0.002100754**
GPC6	1.000148643	0.999940155	1.000357175	0.162315213
NR3C2	0.999905121	0.99907696	1.000733968	0.822413164
PIGR	0.997807919	0.939839479	1.059351799	0.942711015
ROBO2	0.999819932	0.998029994	1.001613079	0.843844085
POU2AF1	0.904301141	0.830425629	0.984748693	**0.020699978**
SPATA18	0.999747641	0.999203135	1.000292443	0.363871432
SLC15A2	1.000104138	0.999704202	1.000504235	0.609859349

**Table 3:** Clinical characteristics and correlations with mRNA expression of AURKA and FAM83A

Characteristic	N=462	AURKA			FAM83A		
		Low (n=231)	High (n=231)	*P* value	Low (n=231)	High (n=231)	*P* value
**Age (years)**				0.277			0.159
<65	202	93	109		91	111	
≥65	250	134	116		134	116	
Unknown	10	4	6		6	4	
**Gender**				**<0.001**			**0.019**
Female	252	147	105		139	113	
Male	210	84	126		92	118	
**Smoking history**				**0.016**			**0.001**
Smoker	317	146	171		142	175	
Non-smoker	145	85	60		89	56	
**T stage**				**0.029**			**0.010**
T1	159	94	65		96	63	
T2	244	112	132		110	134	
T3+T4	56	23	33		23	33	
Unknown	3	2	1		2	1	
**N stage**				**0.032**			**0.004**
N0	298	155	143		163	135	
N1	85	41	44		37	48	
N2+ N3	68	26	42		23	45	
Unknown	11	9	2		8	3	
**M stage**				0.437			0.580
M0	311	153	158		152	159	
M1	24	9	15		10	14	
MX	124	67	57		67	57	
Unknown	3	2	1		2	1	

**Table 4:** Reactome of AURKA and FAM83A

Stable identifier	Pathway
R-HSA-174143	APC/C-mediated degradation of cell cycle proteins
R-HSA-174178	APC/C
R-HSA-8854518	AURKA Activation by TPX2
R-HSA-1640170	Cell Cycle
R-HSA-69278	Cell Cycle, Mitotic
R-HSA-8854050	FBXL7 down-regulates AURKA during mitotic entry and in early mitosis
R-HSA-69275	G2/M Transition
R-HSA-74160	Gene Expression
R-HSA-212436	Generic Transcription Pathway
R-HSA-8854521	Interaction between PHLDA1 and AURKA
R-HSA-392499	Metabolism of proteins
R-HSA-453274	Mitotic G2-G2/M phases
R-HSA-597592	Post-translational protein modification
R-HSA-2565942	Regulation of PLK1 Activity at G2/M Transition
