# Supplementary material for: AURKA and FAM83A are prognostic biomarkers and correlated with Tumor-infiltrating Lymphocytes in smoking related Lung Adenocarcinoma
Source: J Cancer. 2021 Jan 18;12(6):1742–54. doi: 10.7150/jca.51321 (PMC7890332; doi:10.7150/jca.51321)
Supplement: Supplementary file 1 — Supplementary figures and tables. [file jcav12p1742s1.pdf]

Table S1. AUC analysis of any one of 4 DEGs in all LUAD patients.

Gene symbol	AUC	CI.95L	CI.95H	P value
AURKA	0.566	0.514	0.619	0.017
FAM83A	0.585	0.532	0.639	0.002
HSD17B2	0.520	0.466	0.574	0.473
POU2AF1	0.531	0.477	0.585	0.268
AURKA+FAM83A	0.575	0.522	0.627	0.007
AURKA+HSD17B2	0.573	0.520	0.626	0.009
AURKA+POU2AF1	0.587	0.534	0.640	0.002
FAM83A+HSD17B2	0.574	0.520	0.628	0.008
FAM83A+POU2AF1	0.585	0.532	0.639	0.002
HSD17B2+POU2AF1	0.529	0.475	0.583	0.295
AURKA+FAM83A+HSD17B2	0.578	0.525	0.632	0.005
AURKA+FAM83A+POU2AF1	0.592	0.539	0.645	0.001
FAM83A+HSD17B2+POU2AF1	0.576	0.522	0.629	0.006
AURKA+FAM83A+HSD17B2+POU2AF1	0.592	0.538	0.645	0.001

Table S2. AUC analysis of any one of 4 DEGs in early stage LUAD patients (stage I).

Gene symbol	AUC	CI.95L	CI.95H	P value
AURKA	0.571	0.501	0.641	0.059
FAM83A	0.595	0.523	0.666	0.012
HSD17B2	0.548	0.476	0.621	0.200
POU2AF1	0.552	0.479	0.625	0.163
AURKA+FAM83A	0.602	0.533	0.672	0.006
AURKA+HSD17B2	0.591	0.520	0.662	0.016
AURKA+POU2AF1	0.610	0.539	0.681	0.003
FAM83A+HSD17B2	0.621	0.550	0.692	0.001
FAM83A+POU2AF1	0.616	0.545	0.688	0.002
HSD17B2+POU2AF1	0.559	0.486	0.632	0.117
AURKA+FAM83A+HSD17B2	0.621	0.551	0.691	0.001
AURKA+FAM83A+POU2AF1	0.628	0.558	0.699	0.001
FAM83A+HSD17B2+POU2AF1	0.613	0.542	0.685	0.003
AURKA+FAM83A+HSD17B2+POU2AF1	0.631	0.561	0.701	0.000

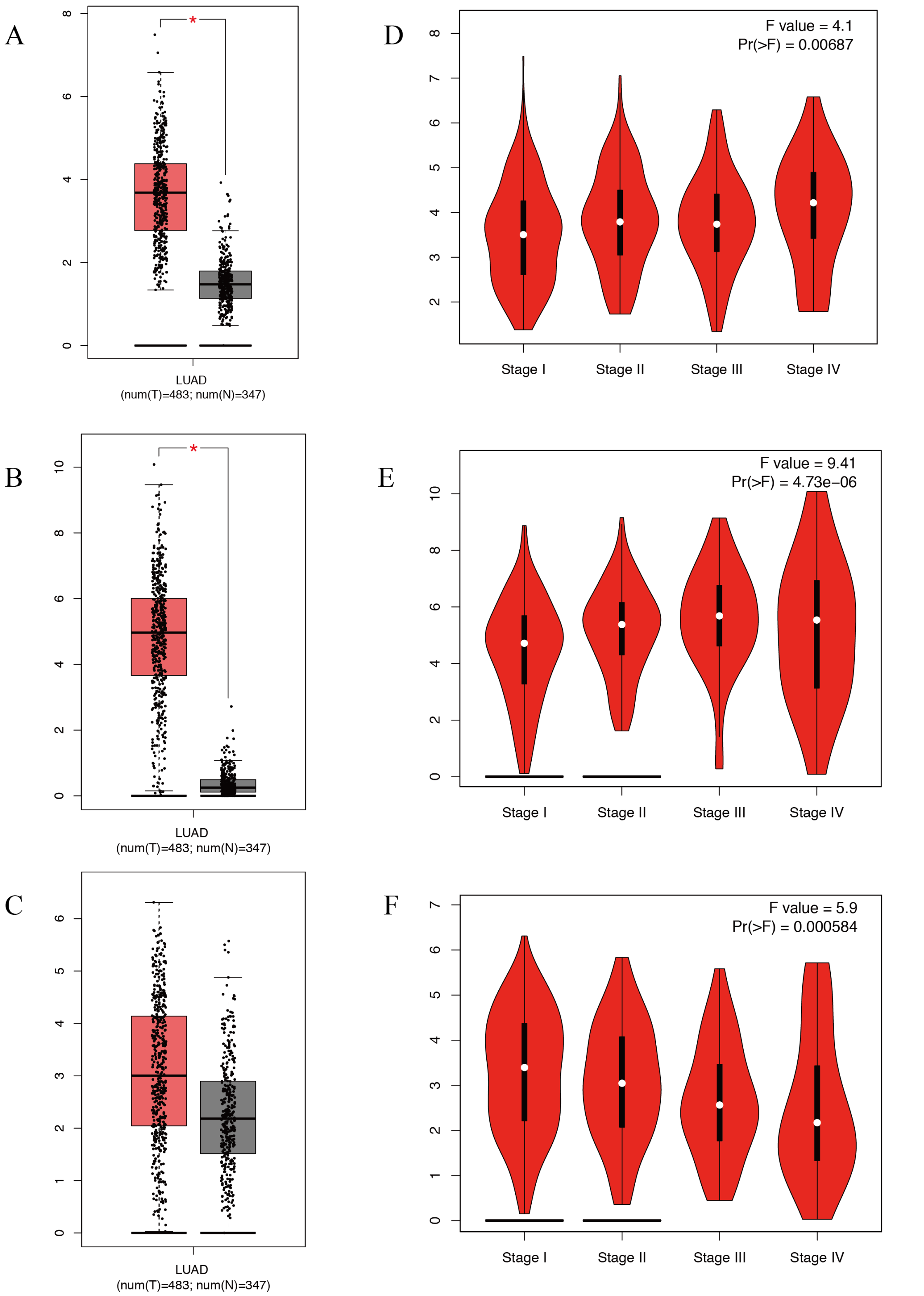

Figure S1. Gene expression levels in LUAD patients according to online tools GEPIA. (A) AURKA expression levels between LUAD and normal lung tissues. (B) AURKA expression levels among LUAD patients in different stages. (C). FAM83A expression levels between LUAD and normal lung tissues. (D) FAM83A expression

levels among LUAD patients in different stages. (E) POU2AF1 expression levels between LUAD and normal lung tissues. (F) POU2AF1 expression levels among LUAD patients in different stages.

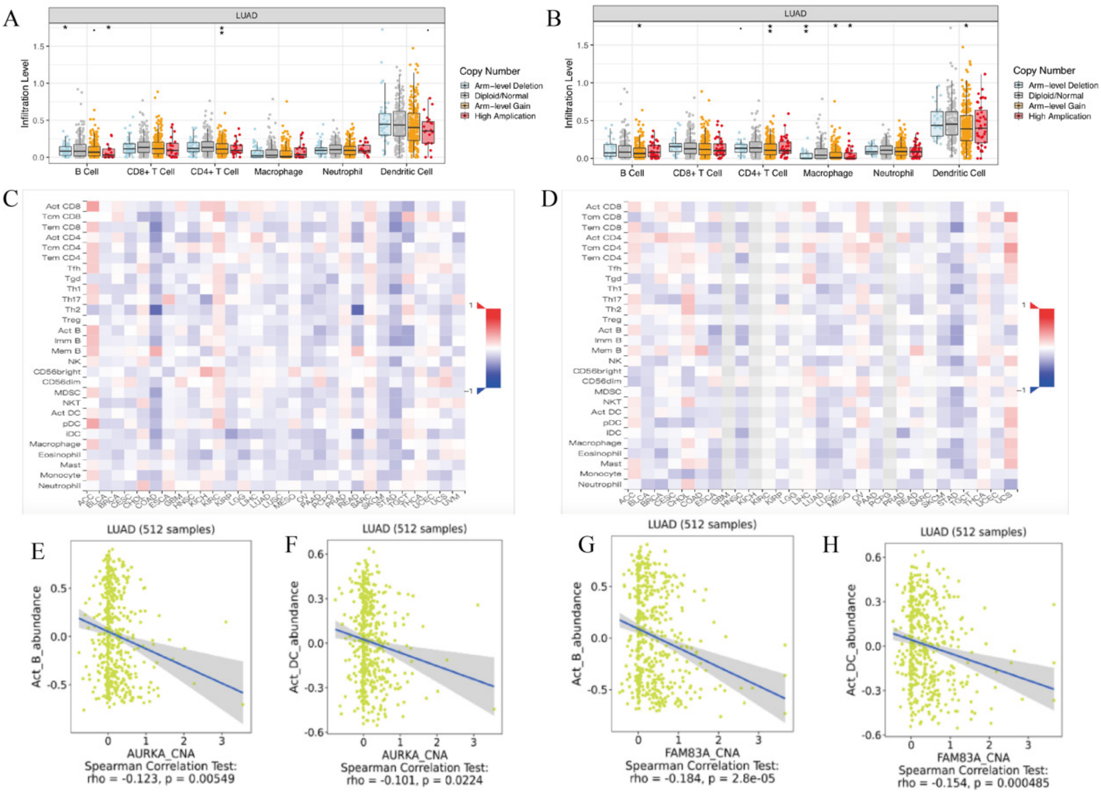

Figure S2. (A) Correlation between somatic copy number variation (SCNA) level of AURKA and immune cell infiltration level. (B) Correlation between SCNA level of FAM83A and immune cell infiltration level. (C) Heatmap analysis of SCNA level of AURAK and tumor infiltrating lymphocytes. (D) Heatmap analysis of SCNA level of FAM83A and tumor infiltrating lymphocytes. (E) Correlation between SCNA level of AURAK and B Cell infiltration level. (F) Correlation between SCNA level of AURAK and Dendritic Cell infiltration level. (G) Correlation between SCNA level of FAM83A and B Cell infiltration level. (H) Correlation between SCNA level of FAM83A and Dendritic Cell infiltration level.

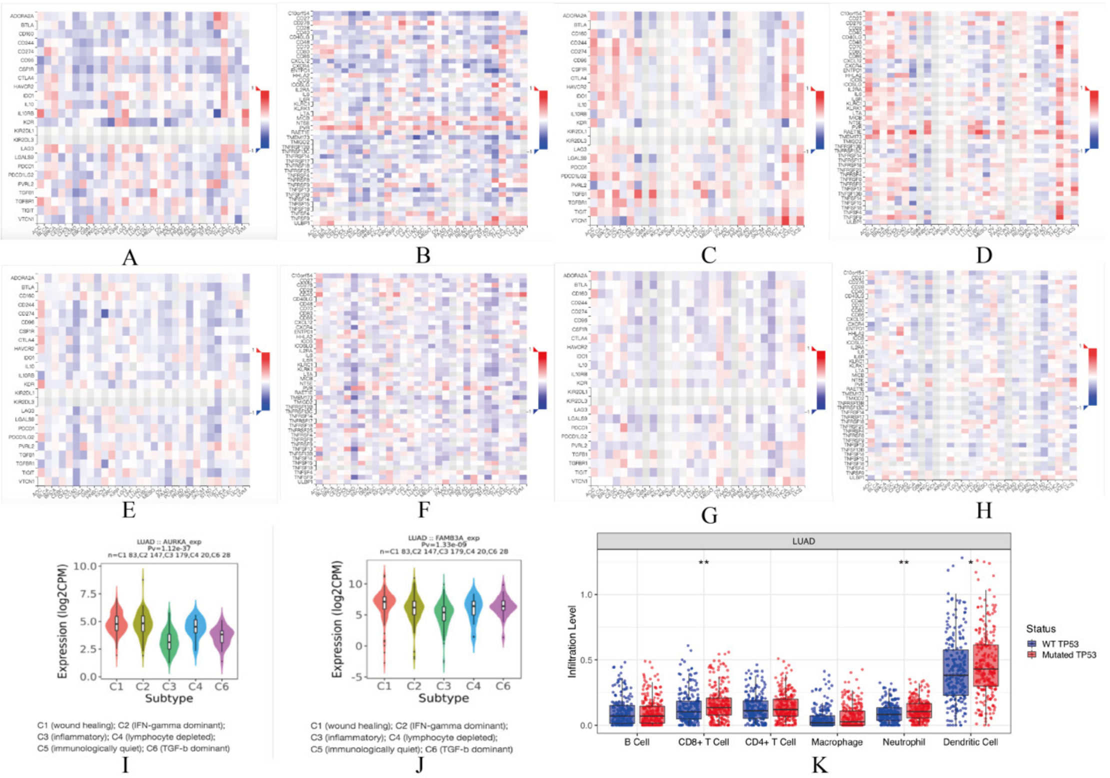

Figure S3. (A) Heatmap analysis of AURAK expression level and immunoinhibitors. (B) Heatmap analysis of AURAK expression level and immunostimulators. (C) Heatmap analysis of FAM83A expression level and immunoinhibitors. (D) Heatmap analysis of FAM83A expression level and immunostimulators. (E) Heatmap analysis of SCNA level of AURAK and immunoinhibitors. (F) Heatmap analysis of SCNA level of AURAK and immunostimulators. (G) Heatmap analysis of SCNA level of FAM83A and immunoinhibitors. (H) Heatmap analysis of SCNA level of FAM83A and immunostimulators. (I-J) AURKA and FAM83A expression distribution across LUAD subtype. (K) TP53 mutation related to CD8+ T Cell, Neutrophil and Dendritic Cell infiltration level.
